# Cryopreservation of dog semen in a Tris extender with two different 1% soybean preparations compared with a Tris egg yolk extender

**DOI:** 10.1002/vms3.445

**Published:** 2021-02-11

**Authors:** Ulrika Hermansson, Anders Johannisson, Eva Axnér

**Affiliations:** ^1^ University Animal Hospital Uppsala Sweden; ^2^ Division of Reproduction Department of Clinical Sciences Faculty of Veterinary Medicine and Animal Science Swedish University of Agricultural Sciences SLU Uppsala Sweden

**Keywords:** canine, extender, reproduction, reproductive tract, semen freezing, soybean lecithin

## Abstract

Egg yolk is widely used as a cryoprotectant in dog semen extenders, but there is a risk of contamination with animal pathogens. In addition, egg yolk may vary in composition, making it difficult to standardize the extender. Lecithin is an animal protein‐free alternative to egg yolk for semen cryopreservation. Recently, it was shown that 1% of soybean lecithin type II‐S was better than 2% for freezing canine semen. The aim of the study was to compare two different types of soybean lecithin, with egg yolk as a control. Ejaculates from eight dogs were divided into three equal parts and diluted with a Tris‐based extender, containing either 20% egg yolk, 1% soybean lecithin Type II‐S or 1% soybean lecithin Type IV‐S. The samples were then frozen. Sperm motility was evaluated by computer‐assisted sperm analysis (CASA), acrosome integrity (FITC‐PNA/PI) and sperm membrane integrity (SYBR‐14/PI) post‐thaw, as well as after 2 and 4 hr incubation at 37°C. Post‐thaw sperm chromatin structure assay and plasma membrane integrity were evaluated by flow cytometry. Total motility, sperm plasma membrane integrity and acrosome integrity were significantly better in the egg yolk extender than in the two soybean lecithin‐based extenders. Individual motility post‐thaw differed more than in the fresh samples, illustrating individual differences in tolerance to the cryostress. The DNA Fragmentation Index (% DFI) was significantly lower in the Tris egg yolk (TEY) extender compared to any of the soybean‐based extenders. The number of high green stained spermatozoa were significantly higher in Type IV‐S compared to the control TEY extender. In conclusion, egg yolk was superior to the two lecithin‐based extenders to cryopreserve canine semen.

## Graphical abstract

Lecithin is an animal‐protein‐free alternative to egg yolk for semen cryopreservation. The aim of the study was to compare two different types of soybean lecithin, with egg yolk as a control. Egg yolk was superior to the two lecithin‐based extenders in this study.

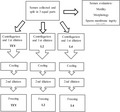

## 1 INTRODUCTION

The ability to cryopreserve semen has made it possible to exchange genetic material over long distances, and enable long time storage. Egg yolk is widely used as a cryoprotectant in dog semen extenders but there are some concerns and risks with the use of egg yolk including a risk of bacterial contamination and a potential risk of causing disease (Bousseau et al., 1998). Egg is a biological product and the composition of egg yolk may be variable, making it difficult to standardize extenders that include egg yolk (Bousseau et al., 1998). Soybean lecithin can replace egg yolk in semen extenders. Lecithin is a phospholipid fraction that can substitute for high molecular weight lipoprotein and phospholipids in egg yolk (Layek et al., 2016). Soybean lecithin has been used in numerous species for semen cryopreservation, including dog (Axner & Lagerson, 2016; Beccaglia et al., 2009; Dalmazzo et al., 2018; Hidalgo et al., 2014; Nöthling et al., 2007; Sanchez‐Calabuig et al., 2017). Different protocols have been used and the first method described by Nelson et al. (1976) was modified by Singh et al., (2012). Cryopreservation of dog semen has been performed with 0.04% or 1.5% soybean lecithin (Beccaglia et al., 2009; Hidalgo et al., 2014), respectively. Both of these studies report that post‐thaw sperm quality was similar to freezing in a Tris egg yolk (TEY) extender. In a study by Axnér and Lagerson (2016), it was concluded that an extender with 1% soybean lecithin extender resulted in better post‐thaw sperm motility than 2% soybean lecithin for freezing dog semen. The aim of this study was to compare two tris‐based extenders containing 1% soybean lecithin from two different sources: type II‐S (Sigma P5638) which was used by Axnér and Lagerson (2016), and type IV‐S (Sigma P3644) that had previously yielded similar results as an egg yolk extender for the cryopreservation of goat (Salmani et al., 2014) and rabbit spermatozoa (Nishijima et al., 2015). A TEY extender was used as a control for the cryopreservation of dog semen.

## 2 MATERIALS AND METHODS

### 2.1 Animals and initial semen quality

Six privately owned purebred dogs of medium and large size (Labrador retriever, White German Shepherd, Smooth collie, Australian Kelpie and Rottweiler), and two Beagles belonging to the Swedish University of Agricultural Sciences, were included in the study. Their ages varied between 2 and 6 years. Semen was collected by digital manipulation into a pre‐warmed plastic vial. Inclusion criteria were that the ejaculates should have ≥70% motile spermatozoa, ≥70% morphologically normal spermatozoa and total sperm numbers ≥600 × 10^6^.

### 2.2 Ethical approval

This work followed established internationally recognized high standards (best practice) of individual veterinary clinical patient care. Dog owners were informed about the procedures and signed a written consent before collecting semen from their dog. Ethical approval from a committee was not required for the procedures involved in the study.

### 2.3 Experimental design

**FIGURE 1:**
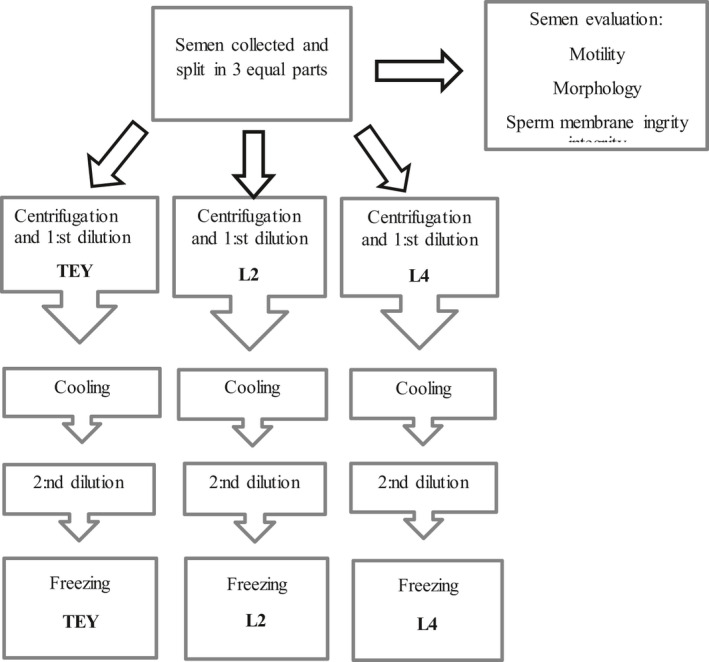
Experimental design. Semen collection and freezing. The procedure was repeated for each ejaculate. *N* = 8 dogs

**FIGURE 2:**
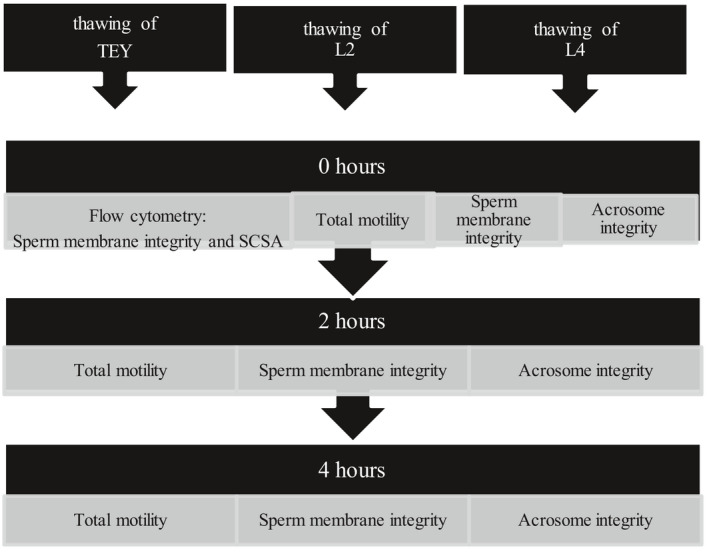
Experimental design. Thawing and semen evaluation. The procedure was repeated for each ejaculate. *N* = 8 dogs

For an overview of the experimental design see Figures 1 and 2.

### 2.4 Extenders

A TEY extender routinely used for cryopreservation at our clinic was used as a control. Control extenders and thawing media were prepared according to the protocol described by Linde‐Forsberg (2014). The composition of the control extender was (per 100 ml): 3.025 g Tris (Merck Eurolab AB), 1.7 g citric acid (Sigma), 1.25 g fructose (Kebo‐Lab), 3 ml glycerol (Kebo‐Lab), 20 ml egg yolk, 0.06 g Na‐benzylpenicillin (Boehringer Ingelheim Vetmedica) and 0.1 g streptomycin sulphate (Sigma‐Aldrich^®^, Inc.). The pH of all extenders was approximately 6.75. The control extender, used for the second dilution had the same composition except that it contained 7 ml glycerol and 1% (v/v) Equex STM paste (Nova Chemical Sales, Scituate, Inc.). The lecithin extenders were based on the same recipe but with egg yolk replaced by 1% soybean lecithin from two different sources; type II‐S, Sigma P5638 (L2) or type IV‐S, Sigma P3644 (L4), and no Equex in the second extenders. Equex STM paste was omitted from the lecithin extenders as it is thought to exert its effect by modifying the egg yolk. Therefore, it is not likely to have a positive effect in an extender without egg yolk. This was confirmed by the absence of post‐thaw sperm motility when Equex was added to a lecithin extender for cryopreservation of dog spermatozoa (Beccaglia et al., 2009).

The soybean lecithin extenders were mixed, centrifuged at 2,900 g for 15 min and thereafter pH was adjusted and the supernatant solution passed through 11‐µm and 0.45‐µm filters. The extenders were divided into equal‐sized aliquots and stored at −18°C until use.

The thawing media had the same composition as the extenders used for freezing but without egg yolk, soybean lecithin and Equex STM paste. Bovine serum albumin (2% w/t vol, Sigma A 4919) was added to the thawing extender for all treatments just before use to prevent agglutination of spermatozoa in the soybean lecithin extenders.

### 2.5 Semen freezing

The semen was frozen using the Uppsala method (Linde‐Forsberg, 2014), with the same cooling and freezing rates as previously described for this method (Peña & Linde‐Forsberg, 2000). One ejaculate from each dog was divided into three parts with equal sperm numbers, centrifuged at 700 g for 6 min and the supernatant was removed. Each pellet was diluted at room temperature with one of three Tris‐based extenders containing 20% egg yolk, 1% soybean lecithin type II‐S, (Sigma P5638) or 1% soybean lecithin type IV‐S (Sigma P3644) to a concentration of 400 × 10^6^ spermatozoa/ml. The sperm sample was placed in a bench cooler at room temperature for 75 min and thereafter diluted a second time to a final sperm concentration of 200 × 10^6^ spermatozoa/ml at 4°C. The extenders used for the second dilution step had the same composition as the first extenders but with a higher glycerol concentration and the addition of Equex STM paste to the egg yolk extender.

Extended semen was loaded in 0.5 ml straws and frozen as previously described by Rota et al. (1997). In brief, straws were put in a goblet and the goblets at the top of a cane. The cane was put in a canister which was then frozen by lowering it vertically, in three steps, in an Apollo SX‐18 LN, tank (MVE Cryogenetics B) containing 15–18 cm of liquid nitrogen for 2, 2 and 1 min, the top of the goblets being at 7, 13 and 20 cm below the opening of the tank, respectively.

Thawing was done in a water bath at 37°C for 60 s and each straw was immediately extended in 0.5 ml thaw media at 37°C and incubated at 37°C for 5 min (time 0), 2 hr and 4 hr before evaluation.

### 2.6 Semen evaluation

#### 2.6.1 Fresh semen

After each semen collection, the volume and colour of the ejaculate were evaluated. Subjective motility was evaluated under a phase‐contrast microscope at ×200 and ×400 magnification. Sperm concentration was determined using a photometer calibrated for canine spermatozoa (Spermacue Minitüb). The total number of spermatozoa in each ejaculate was calculated. Morphology was evaluated by fixing a drop of fresh semen with a drop of formol‐saline; 100 spermatozoa were evaluated at ×400 magnification using a phase‐contrast microscope. Five microlitres of fresh semen was smeared onto a microscope slide for evaluation of acrosome integrity.

#### 2.6.2 Post‐thaw semen evaluation

Semen was evaluated for motility, acrosome integrity [fluorescein isothiocyanate (FITC)‐peanut agglutinin (PNA)/propidium iodide (PI)] and sperm membrane integrity (SYBR‐14/PI) at time 0 (after 5 min incubation at 37°C), 2 and 4 hr after incubation at 37°C. Flow cytometry analyses of sperm membrane integrity and sperm chromatin integrity were performed at time 0.

#### 2.6.3 Computer‐assisted sperm analysis

Sperm motility was evaluated with a SpermVision analyser (Minitüb GmbH). Five microlitres of semen samples was pipetted on to a warm microscope slide and a coverslip placed on top. Sperm motility was analysed in eight fields using a software program (SpermVision) with settings adjusted for dog spermatozoa. Total motility and the following parameters were evaluated: VCL (track velocity), VAP (path velocity), VSL (straight line velocity), LIN (linearity), STR (straightness), WOB (wobble), BCF (beat cross frequency) and ALH (amplitude of lateral head displacement).

#### 2.6.4 Sperm membrane integrity and acrosome integrity

Integrity of sperm membranes (viability) and acrosomal integrity were evaluated with an epifluorescent microscope (Laborlux‐11; Leitz) with a UV ParaLens® adapter (Becton Dickinson) at ×400 magnification.

For evaluation of sperm membrane integrity, 15 µl semen was mixed with 5 µl PI (340 µM) and 5 µl SYBR‐14 to a final concentration of 2 µM and incubated in 37°C for 15 min (Molecular Probes). After incubation, a 5 µl aliquot of stained semen was placed on a warm microscope slide and coverslip was placed on top. Two hundred spermatozoa were evaluated. Live spermatozoa stained green with SYBR‐14 and dead spermatozoa stained red with PI. Moribund spermatozoa stained both green and red.

An aliquot of 5 µl sperm suspension was smeared on to a microscope slide and allowed to air dry. The sperm membranes were then permeabilized with 95% ethanol for 30 s. Ninety microliters of FITC‐PNA (100 µg/ml in PBS) was mixed with 5 µl of PI (340 µM in PBS, final concentration 18 µM) and 20 µl of this mixture was spread over each smear. The slides were incubated in a moist chamber at 4°C for 30 min, then rinsed with distilled water and air‐dried at 4°C (Axnér et al., 2004). The slides were mounted with 10 µl antifade solution. A coverslip was placed on each smear and sealed with nail polish; 200 spermatozoa were assessed in each smear and classified as having an intact, damaged or reacted/missing acrosome.

#### 2.6.5 Flow cytometry

##### Sperm membrane integrity

Sperm membrane integrity was also assessed by flow cytometry using SYBR14‐PI staining (Garner et al., 1994). The thawed semen samples with a concentration of approximately 0.5 × 10^6^ spermatozoa/ml were diluted using Buffer B (JM Morrell, SLU, patent pending); 300 µl of these sperm suspensions was stained with 0.6 µl SYBR‐14 (final concentration 0.02 µm) and 3 µl of PI (final concentration 12 µm), both from Live‐Dead Sperm Viability Kit L‐7011; (Invitrogen) and incubated for 10 min at 38°C. The stained samples were evaluated with a FACSVerse flow cytometer (Beckon Dickinson) with excitation with a blue laser (488 nm). Forward (FSC) and side (SSC) scatter were used to exclude debris particles. The green fluorescence (FL1) was detected with a 527/32 nm band‐pass filter, whereas the red fluorescence (FL3) was measured using a 700/54 nm band‐pass filter. Data from 30,000 events were collected. After gating to exclude non‐sperm events, spermatozoa were classified as being live with intact plasma membrane (SYBR‐14 positive/PI negative), moribund (SYBR‐14 intermediate/PI intermediate) or dead with disaggregated membrane (SYBR‐14 negative/PI positive).

##### Sperm chromatin structure assay

This assay was performed according to the method described previously (Evenson et al., 2002; Morrell et al., 2009) with minor modifications. Briefly, equal volumes of sperm suspension at a concentration of 200 × 10^6^ spermatozoa/ml and Tris‐sodium chloride‐EDTA buffer (0.15 mol/L NaCl, 0.01 mol/L Tris‐HCl, 1 mmol/L EDTA, pH 7.4); (TNE) were mixed together, snap‐frozen in liquid nitrogen (LN_2_) and stored at −80°C until subsequent evaluation by flow cytometry. At the time of analysis, the frozen sperm suspensions were thawed on crushed ice and the sperm concentration was diluted 1:10 with TNE buffer immediately before staining. Subsequently, the spermatozoa were subjected to partial DNA denaturation by mixing 100 µl of sperm suspension with 200 µl of acid‐detergent solution 0.17% Triton X‐100 (Sigma‐Aldrich); (0.15 mol/L NaCl, and 0.08 mol/L HCl; pH 1.2). After 30 s, the spermatozoa were labelled with 600 µl staining solution of acridine orange (Sigma‐Aldrich) (6 μg/ml in 0.1 mol/L citric acid, 0.2 mol/L Na_2_HPO_4_, 1 mmol/L EDTA, 0.15 mol/L NaCl; pH 6.0). Within 3–5 min, the stained samples were evaluated using the flow cytometer (FACSVerse, BDBiosciences). For each sample at least 10,000 events were analysed at a speed of 200 cells/s. Forward scatter (FSC), Side scatter (SSC), FL1 (green fluorescence) and FL3 (red fluorescence) were measured after excitation with a blue laser (488 nm). The DNA Fragmentation Index (%DFI—ratio of the percentage of cells with denatured, single‐stranded DNA to total cells acquired [both with stable, double‐stranded DNA, and denatured single‐stranded DNA]) as well as the percentage of spermatozoa with high green fluorescence, indicating immature spermatozoa, was calculated for each sample using FCS Express version 5 (DeNovo Software).

### 2.7 Statistics

All analyses were made with Minitab 17 (©2013 Minitab Inc.) and SAS 9.2. Parameters with a normal distribution of the residuals were analysed with a repeated‐measures ANOVA in a 3 × 3 factorial design. Fixed factors in the model were time, extender and interaction between treatment and time. Dog was included as a random factor. Pairwise comparisons were made with Tukey's correction. Parameters without a normal distribution of the residuals (VAP, VSL, ALH, LIN and STR) were analysed within time with Friedman's test. For significant results in the Friedman test, pairwise comparisons within time were performed with a Mann–Whitney test.

## 3 RESULTS

### 3.1 Fresh semen quality

The mean total sperm number in individual ejaculates was 978 × 10^6^ spermatozoa (total sperm numbers were between 600 and 2,400 × 10^6^ spermatozoa), and the mean proportion of subjective motility in the fresh semen samples was 85 ± 8% (range 70%–90%). The mean proportion of morphologically abnormal spermatozoa was 14.4 ± 4.2% (range 11%–24%). The mean proportion of spermatozoa with intact acrosomes was 85.4 ± 6.2% (range 78%–95%).

### 3.2 Interactions

Significant interactions between time and extender were found for motility (*p* = 0.013) and VCL (*p* = 0.045) but not for other parameters. Interactions were not tested for data analysed with non‐parametric evaluation (VAP, VSL, ALH, LIN and STR).

### 3.3 Post‐thaw sperm motility and sperm kinetics

**FIGURE 3:**
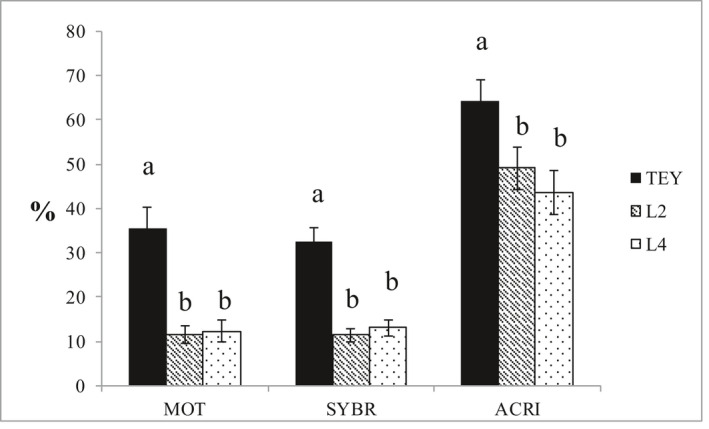
Comparisons of total motility (MOT), membrane integrity (SYBR) and acrosomal integrity (ACRI) among extenders. TEY = Tris egg yolk, L2 = extender with lecithin type II‐S. L4 extender with lecithin type IV‐S. Mean ± SE. *N* = 8. Different letters within parameter indicate significant difference (*p* < 0.05). *N* = 8 dogs

**FIGURE 4:**
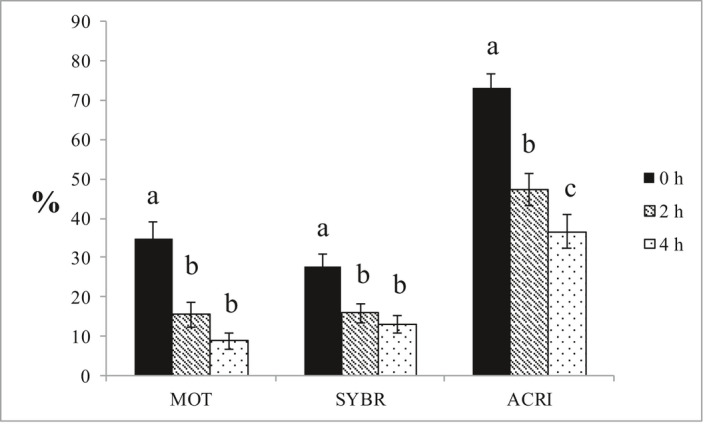
Comparisons of total motility (MOT), membrane integrity (SYBR) and acrosomal integrity (ACRI) among different time points post‐thaw. TEY = Tris egg yolk, L2 = extender with lecithin type II‐S, L4 = extender with lecithin type IV‐S. Mean ± SE. *N* = 8. Different letters within parameter indicate significant difference (*p* < 0.05). Different letters within parameter indicate significant difference (*p* < 0.05). *n* = 8 dogs

Total motility was significantly better in the egg yolk extender than in the two soybean lecithin‐based extenders (*p* < 0.001, Figure 3). Motility decreased significantly over time with a significant interaction between time and treatment (*p* = 0.013) (Figure 4 and Table 1). For the egg yolk extender, there were significant differences between each time point: L2 did not differ with time, whereas L4 differed between time 0 and other time points but not between 2 and 4 hr post‐thaw. At time 4, there were no significant differences between the egg yolk extender and either of the soybean lecithin extenders. There were no significant differences between the soybean lecithin‐based extenders at any time post‐thaw. Individual motility post‐thaw differed more than in the fresh samples, illustrating individual differences in tolerance to the cryostress.

**TABLE 1:** Pairwise comparisons of total motility, sperm membrane integrity and acrosome integrity in different extenders at different incubation times

Time	Total motility			Sperm membrane integrity			Acrosome integrity		
	TEY	L2	L4	TEY	L2	L4	TEY	L2	L4
0	56.6 ± 6.7^Aa^	22.0 ± 3.3^Ab^	26.1 ± 4.3^Ab^	45.0 ± 4.7^Aa^	17.1 ± 3.2^b^	21.2 ± 3.4^Ab^	85.6 ± 3.1^A^	66.2 ± 6.8^A^	67.9 ± 5.9^A^
2	33.6 ± 5.9^Ba^	6.1 ± 1.3^Ab^	6.8 ± 0.6^Bb^	29.4 ± 3.9^Ba^	8.8 ± 1.0^b^	9.8 ± 2.0^ABb^	60.3 ± 5.4^B^	43.6 ± 7.0^A^	38.0 ± 6.7^B^
4	15.9 ± 5.3^Ca^	6.5 ± 1.9^Aa^	3.8 ± 1.0^Ba^	22.6 ± 4.9^Ba^	8.4 ± 1.7^b^	8.2 ± 1.7^Bb^	47.2 ± 6.9^B^	37.6 ± 7.8^A^	24.9 ± 5.4^B^

Note

Means ± SE. *N* = 8 dogs.

^AB^Significant differences within column for each parameter.

^ab^Significant differences within row for each parameter (*p* < 0.05).

**FIGURE 5:**
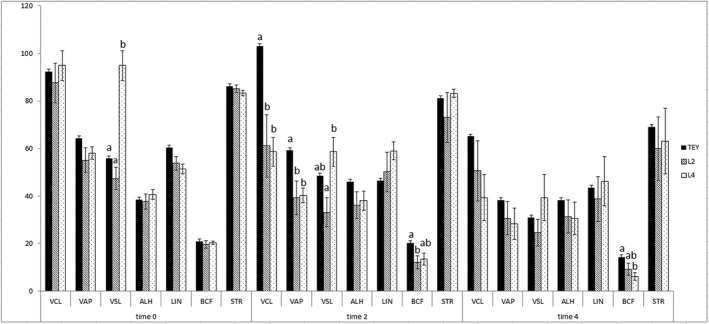
Motility parameters in different extenders. TEY = tris egg yolk extender, L2 = lecithin type II‐S extender, L4 = lecithin type IV‐S extender. Curvilinear velocity VCL (µm/s), average path velocity VAP (µm/s), straight linear velocity VSL (µm/s), average lateral head displacement ALH (µm × 10), linearity LIN (%), Beat cross frequency BCF (Hz), Straightness STR (%).^abc^Different letters indicate significant differences (*p* < 0.05) between treatments within time points. Mean ± SE. *N* = 8 dogs

Sperm motility parameters are presented in Figure 5. At time 0, only VSL differed between treatments with significantly higher VSL for the L4 extender than the two other extenders. At time 2 hr post‐thaw, VSL differed only between the two lecithin extenders with higher VSL for the L4 extender; at time 4 hr post‐thaw there were no differences in VSL among the extenders. At time 2 hr post‐thaw, VCL and VAP were higher in TEY than in either of the lecithin extenders, whereas BCF was higher in TEY than in the L2 extender at 2 hr post‐thaw and higher than in the L4 extender at 4 hr post‐thaw.

### 3.4 Post‐thaw sperm membrane integrity

Sperm plasma membrane integrity was significantly better in the TEY than in either of the two soybean lecithin‐based extenders at any given time (*p* < 0.001, Figure 3, Table 1). There were no significant differences between the two soybean lecithin‐based extenders at any given time. Time was a significant factor in the overall comparison and in pairwise comparisons for TEY and the L4 extender (Figure 4 and Table 1).

### 3.5 Post‐thaw acrosome integrity

Acrosome integrity was significantly better in the TEY compared to the soybean lecithin extenders over time (*p* < 0.005), but there were no significant differences in pairwise comparisons at any given time point There was an overall effect of time with a significant decrease in acrosomal integrity between all the time points (Figure 4, Table 1).

### 3.6 Post‐thaw sperm chromatin integrity

**FIGURE 6:**
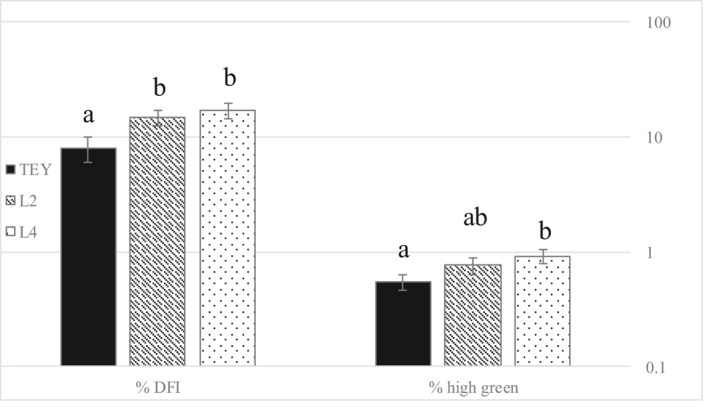
Sperm chromatin structure assay (SCSA) immediately after thawing. DNA Fragmentation Index (%DFI) and % of spermatozoa with high green fluorescence indicating immature spermatozoa. TEY = Tris egg yolk, L2 = extender with lecithin type II‐S, L4 = extender with lecithin type IV‐S. Different letters within parameter indicate significant difference (*p* < 0.05). Mean ± SE. *N* = 8 dogs

The % DFI was significantly lower in TEY compared to either of the soybean‐based extenders. There was no significant difference between the soybean‐based extenders (Figure 6). The proportion of high green stained spermatozoa was significantly higher in L4 compared to the control TEY extender. There were no significant differences between the soybean‐based extenders, or between the control TEY and L2 (Figure 6).

### 3.7 Post‐thaw sperm membrane integrity (flow cytometry)

The flow‐cytometric evaluation confirmed the data from fluorescence microscopy. Samples frozen in TEY had significantly better sperm plasma membrane integrity than samples frozen with either of the soybean‐based extenders. There were no significant differences between the soybean‐based extenders in sperm plasma membrane integrity.

## 4 DISCUSSION

The results in this study showed that motility, acrosome integrity and membrane integrity deteriorated post‐thaw and with time, regardless of extender used. However, the deterioration was greater in the soy lecithin‐based extenders, revealing that egg yolk was superior in all evaluated post‐thaw parameters to the two different soy lecithin extenders. Replacing egg yolk with type IV‐S lecithin gave similar results to the type II‐S lecithin that was previously compared with egg yolk (Axnér and Lagerson, 2016).

Only VSL (straight line velocity) differed between the two lecithin extenders and was significantly higher in the L4 compared to the L2 extender. Higher VCL, VAP and BCF in the egg yolk extender and a higher VSL in the extender with L4 might indicate a faster but less linear motion in TEY and a faster linear motility in spermatozoa extended in L4 than in TEY or L2.

The mean total motility at time 0 was relatively low in all extenders, including the control (Table 1). The control extender is routinely used clinically in our laboratory with an expected post‐thaw motility >50% in ejaculates with fresh semen parameters above the reference limits for normal. Two of the dogs had a post‐thaw motility <50% (47 and 22%) in the control extender which affected the mean values (Figure 3a). Their results were included in the study as our inclusion criteria only related to fresh semen quality and it is possible that different extenders might vary in their effects on ejaculates from different individuals. In the lecithin extenders, however, all samples had <50% motility immediately post‐thaw, further indicating that the lecithin extenders would be unsuitable for dog semen freezing both on individual and general level.

The type IV‐S that is extracted from type II‐S has higher phosphatidylcholine content than the type II‐S. Both soybean extenders contained 1% (wt/vol) soybean lecithin extract but because the composition of lecithin type IV‐S differs from type II‐S we might have expected some differences between the two extenders. Both types have been used for cryopreservation of mammalian spermatozoa but, to our knowledge, they have not been compared (Nishijima et al., 2015; Papa et al., 2011; Salmani et al., 2014; Tarig et al., 2017; Yildiz et al., 2013).

Chromatin fragmentation (% DFI) was significantly lower in TEY compared to any of the soybean‐based extenders. It is caused by a number of different factors, such as oxidative stress (Aitken & De Iuliis, 2007), alterations to chromatin remodelling during spermiogenesis (Carrell et al., 2007), apoptosis of germ cells at the beginning of meiosis (Sakkas et al., 2004), and others. The % DFI can be directly associated with fertility in humans (Zhu & Liu, 2000), whereas for bulls the value of %DFI for fertility prediction may depend on the breed (Morrell et al., 2017).

The percentage of high green stained spermatozoa was significantly higher in L4 compared to TEY. High green stainings are believed to represent immature spermatozoa. There might be a correlation between % DFI and high green as incompletely condensated chromatin might be more susceptible to DNA strand breaks (Revay et al., 2009).

Results from studies on soybean lecithin extenders for cryopreservation of dog spermatozoa are inconclusive, with contradictory results. Beccaglia et al. (2009), using a very low soybean concentration of 0.04%, had similar motility, number of capacitated sperm and number of spermatozoa bound to the ZP with the lecithin extender as in TEY. Dalmazzo et al. (2018), also using low concentrations (0.01%–0.1%), also reported similar results between egg yolk and soybean lecithin extenders for dog sperm cryopreservation. It is difficult, however, to compare the results by Dalmazzo et al. (2018) with the results from our study as the average post‐thaw motility in all their tested extenders, including the control, was below 20%. In the study by Dalmazzo et al. (2018), higher concentrations of lecithin types FP40 and 8160 (0.05%–0.1%) were deleterious. In contrast, Hidalgo et al. (2014) reported that 1.5% soybean lecithin in the extender resulted in similar post‐thaw sperm viability and acrosome integrity as egg yolk. Motility and lecithin type were not reported in their study (Hidalgo et al., 2014). Other studies, however, showed inferior results when ejaculated or epididymal dog spermatozoa were cryopreserved in a soybean lecithin extender compared with an egg yolk extender (Nöthling et al., 2007; Sanchez‐Calabuig et al., 2017), which are similar to our results. From the published results it is thus difficult to draw any conclusions about the optimal concentrations of soybean lecithin for dog semen cryopreservation or if the optimal concentration might vary with lecithin type.

Some studies have indicated that soybean lecithin is an antioxidant, and better results are obtained when lecithin samples contain a high concentration of phosphatidylcholine (Judde et al., 2003; Nasir et al., 2007). The exact amount of phosphatidylcholine is not reported in most studies, but might be a contributing factor.

Dalmazzo et al. (2018) suggest that a too high concentration of phosphatidylcholine in some of the extenders they used might have caused decreased mitochondrial activity and, therefore, decreased motility. Corresponding results were found by Del Valle et al. (2012) also suggesting that lecithin could promote a decrease in mitochondrial function in the ram. Del Valle et al. (2012) suggested that lecithin could alter the inner mitochondrial membrane.

There is a huge variation in the lecithin‐based extenders used in previous studies, making it difficult to compare them with each other, for instance how much phosphatidylcholine they contain. The control extenders with egg yolk may vary in composition and might not be fully comparable. In conclusion, egg yolk was superior to either of the two soybean lecithin‐based extenders used in this study. However, other authors show promising results with soybean lecithin and more studies are needed. More effort should be made to find out which soybean lecithin concentration and concentration of phosphatidylcholine is best for freezing dog spermatozoa, and various functional tests should be used to predict sperm fertility.

## AUTHOR CONTRIBUTIONS

Ulrika Helena Hermansson: Conceptualization; Investigation; Methodology; Writing‐original draft. Anders Johannisson: Data curation; Methodology; Software; Writing‐review & editing. Eva Axnér: Conceptualization; Data curation; Formal analysis; Methodology; Supervision; Writing‐review & editing.

### PEER REVIEW

The peer review history for this article is available at https://publons.com/publon/10.1002/vms3.445.
